# Emerging Role of Platelet-Endothelium Interactions in the Pathogenesis of Severe SARS-CoV-2 Infection-Associated Myocardial Injury

**DOI:** 10.3389/fimmu.2022.776861

**Published:** 2022-02-04

**Authors:** Theresa M. Rossouw, Ronald Anderson, Pravin Manga, Charles Feldman

**Affiliations:** ^1^ Department of Immunology, Faculty of Health Sciences, University of Pretoria, Pretoria, South Africa; ^2^ Department of Internal Medicine, Faculty of Health Sciences, University of the Witwatersrand, Johannesburg, South Africa

**Keywords:** ACE2 receptor, acute myocardial injury, cardiovascular disease, corona virus disease (COVID-19), endotheliitis, nucleocapsid (N) protein, platelet activation, spike protein

## Abstract

Cardiovascular dysfunction and disease are common and frequently fatal complications of severe acute respiratory syndrome coronavirus 2 (SARS-CoV-2) infection. Indeed, from early on during the SARS-CoV-2 virus pandemic it was recognized that cardiac complications may occur, even in patients with no underlying cardiac disorders, as part of the acute infection, and that these were associated with more severe disease and increased morbidity and mortality. The most common cardiac complication is acute cardiac injury, defined by significant elevation of cardiac troponins. The potential mechanisms of cardiovascular complications include direct viral myocardial injury, systemic inflammation induced by the virus, sepsis, arrhythmia, myocardial oxygen supply-demand mismatch, electrolyte abnormalities, and hypercoagulability. This review is focused on the prevalence, risk factors and clinical course of COVID-19-related myocardial injury, as well as on current data with regard to disease pathogenesis, specifically the interaction of platelets with the vascular endothelium. The latter section includes consideration of the role of SARS-CoV-2 proteins in triggering development of a generalized endotheliitis that, in turn, drives intense activation of platelets. Most prominently, SARS-CoV-2–induced endotheliitis involves interaction of the viral spike protein with endothelial angiotensin-converting enzyme 2 (ACE2) together with alternative mechanisms that involve the nucleocapsid and viroporin. In addition, the mechanisms by which activated platelets intensify endothelial activation and dysfunction, seemingly driven by release of the platelet-derived calcium-binding proteins, SA100A8 and SA100A9, are described. These events create a SARS-CoV-2–driven cycle of intravascular inflammation and coagulation, which contributes significantly to a poor clinical outcome in patients with severe disease.

## Introduction

Coronavirus disease-19 (COVID-19), caused by the novel coronavirus, severe acute respiratory syndrome coronavirus 2 (SARS-CoV-2), is a multi-system inflammatory disorder, which, in its severe form, is characterized by endothelial damage and hypercoagulability (1). Although the etiology of the arterial and venous thromboembolic events observed in 10-25% of patients admitted with SARS-CoV-2 infection (2) is still being debated, the role of the complex interactions between platelets, the endothelium and SARS-CoV-2 are increasingly recognized. COVID-19-associated thrombosis has been reported in many sites, such as the skin (3), respiratory tract (4, 5), brain (6), gastrointestinal tract (7) and extremities (8), but is particularly catastrophic when the heart is involved (9), especially in the context of a pro-atherosclerotic milieu.

From early on in our understanding of infection with this coronavirus, it has been recognized that underlying cardiac comorbid conditions are important risk factors for COVID-19 infection and that cardiac complications may occur, even in patients with no underlying cardiac disorders, as part of the acute infection. One of the early publications from Wuhan, describing 41 hospitalized cases with COVID-19 pneumonia, noted that 73% of the patients were men, with 32% having underlying co-morbidities, including hypertension in 15% and cardiovascular diseases in 15% (10). A number of complications were noted in the patients, including acute cardiac injury in 12%. Shortly thereafter, several reports confirmed that underlying cardiovascular co-morbidities, as well as cardiac complications due to acute COVID-19 infection, were associated with more severe disease and increased morbidity and mortality (11–16). From these collective publications, it was noted that the most common cardiac complication was acute cardiac injury, defined by significant elevation of cardiac troponins. The acute cardiac injury was secondary to a number of cardiac conditions, including acute myocardial infarction due to acute coronary syndrome (ACS), myocarditis, arrhythmias, and cardiomyopathy, while cardiogenic shock and cardiac arrest were also reported. Thromboembolic disease, both venous and arterial, was also described. The potential mechanisms of cardiovascular complications included direct viral myocardial injury, systemic inflammation induced by the virus, sepsis, arrhythmia, myocardial oxygen supply-demand mismatch, electrolyte abnormalities, and hypercoagulability (17–21). As was noted in 2020, despite many publications there remain many uncertainties regarding the origins of these cardiac complications (22).

The aim of the current manuscript is to review recent data on the clinical manifestations and mechanisms of acute cardiac involvement in patients with SARS-CoV-2 infection. We specifically address acute myocardial injury and ACS, and the potential mechanisms of these complications, especially with regard to the role of platelet-endothelium interactions, concentrating on publications that appeared mainly during 2020/2021.

## The Clinical Aspects of COVID-19 and Cardiovascular Disease

While underlying cardiovascular comorbidities are a risk factor for COVID-19 infection and severity, the infection itself has the potential to cause a significant impact on the cardiovascular health of millions of people worldwide. The virus can infect the heart, as well as vascular tissue, circulating cells, and various organs, through its interaction with the ACE2 receptor, which it uses as a host cell receptor through its spike protein. It is, therefore, not surprising that cardiovascular events are a common extra-pulmonary manifestation of COVID-19 infection (extensively reviewed in 17 and 18). The potential cardiac manifestations of COVID-19 infection are shown in **Table 1**, with the putative mechanisms depicted in **Table 2** (17–21).

**Table 1:** Potential cardiac complications in COVID-19 infections*.

• Acute myocardial injury – elevated troponin levels
• Acute coronary syndrome
○ Non-ST-elevation acute coronary syndrome – NSTEMI or unstable angina
○ ST elevation myocardial infarction (STEMI)
○ Acute myocardial infarction
▪ *Type 1 – acute atherothrombotic coronary artery disease, mostly plaque rupture or thrombosis*
▪ *Type 2 – oxygen supply-demand mismatch*
• Cardiomyopathy
○ Include stress cardiomyopathy – Takotsubo cardiomyopathy
• Myocarditis
• Arrhythmia
• Drug-induced cardiac effects
• Cardiac failure
• Hypotension and/or cardiogenic shock
• Cardiac arrest

*See References (17–21).

**Table 2:** Possible causes of cardiac events in COVID-19 infection*.

• Direct cardiac injury
○ Viral-induced myocardial injury
○ Tropism of the virus for the ACE-2 receptor
• Systemic inflammation—cytokine release syndrome and multisystem inflammatory syndrome
• Sepsis
• Plaque rupture or thrombosis
• Hematological changes—arterial thrombosis, hypercoagulability/coagulopathy and disseminated intravascular coagulation
• Hypoxia
•Myocardial oxygen supply-demand mismatch
• Dysfunctional endothelial response
• Arrhythmia
• Electrolyte disorders
• Direct drug toxicity and/or drug-drug interactions

*See References (17–21).

The cardiac manifestations that may occur during COVID-19 infection include acute cardiac injury, myocarditis, acute myocardial infarction (AMI), heart failure, arrhythmia, cardiomyopathy, hypotension, shock, and cardiac arrest (18, 23, 24). An echocardiographic study of patients with COVID-19 documented pre-existing abnormalities, which prompted the authors to suggest caution with regard to attributing such abnormalities to acute COVID-19 infection (25). Among the vascular complications documented are venous and arterial thrombotic events, which are associated with greater need for respiratory support, vasopressors, and hemodialysis, and a longer hospital stay (24, 26). Autopsy studies from patients who died from COVID-19 infection have demonstrated a range of cardiac abnormalities including cardiac hypertrophy and/or enlargement, ventricular dilatation, infarction, and fibrosis (24).

A number of investigators have shown the potential value of a range of cardiac biomarkers, including high sensitivity troponins (Tn; TnT and TnI), creatinine kinase isoenzyme-MB (CK-MB) and N-terminal pro-brain-type natriuretic peptide (BNP; NT-proBNP), either as early indicators of cardiac involvement in COVID-19 infections and/or as markers of disease prognosis (27–34), while measurement of D-dimers is thought to be potentially important in preventing, diagnosing and managing vascular complications (24).

The troponins TnT and TnI are known as the cardiac troponins, and act on cardiac muscle contraction by regulating the calcium-dependent interactions of actin and myosin (35). The CK component of CK-MB is an enzyme that catalyzes the reversible transformation of creatinine and ATP to creatinine phosphate and ADP (35). Lastly, BNP is a hormone produced by the heart, with both BNP and NT-proBNP being released in response to pressure changes within the heart (36). The former two biomarkers indicate myocardial injury, whereas the latter is a marker of hemodynamic stress (37).

In one systematic review and meta-analysis the pooled incidence of elevated levels of TnI, CK-MB and CK in COVID-19 cases were 15.16%, 10.92%, and 12.99%, respectively (38). Another detailed review indicated that troponin levels were elevated in 12% to 28% of cases with SARS-CoV-2 infection (18). While studies have indicated significant differences in biomarker levels in patients with differing COVID-19 infection severity, these levels also depended on whether the biomarkers were measured early on, or later, in the course of the infection (32, 38). Importantly, in COVID-19 cases, particularly those with underlying pre-existing cardiac disease, the prognostic cut-off values of these biomarkers may be lower than the reference standards (39). Furthermore, studies have shown that TnI, CKMB, and Pro-BNP are higher in non-survivors compared with survivors among critically ill patients with COVID-19 infection (28).

Lastly, it is important to be aware that cardiovascular sequelae can occur even in patients with mild COVID-19 infections, as well as in asymptomatic carriers of SARS-CoV-2 who may not even have been tested for this virus, and who may be at risk of developing cardiovascular disorders as bystander effects of the virus (40, 41). It remains essential to recognize and manage cardiovascular events during COVID-19 infection as soon as possible, as they are associated with worse outcomes (26, 42).

### Acute Myocardial Injury

In studies of COVID-19 infection, myocardial injury has been defined as elevation of cardiac troponin I (TnI), or troponin T (TnT) to >99^th^ percentile of the upper reference limit, or the presence of new electrocardiographic or echocardiographic abnormalities (18). Myocardial injury can occur *via* ischemic and non-ischemic mechanisms and the overall rate of acute cardiac injury in older studies has been reported to be between 7.2 and 36%, with rates as high as 59% in those who died (18, 43, 44). The rate of acute cardiac injury has been found to be higher in the case of infection with viruses, such as SARS-CoV-2, that bind the ACE2 receptor, than in those with viruses that do not (45). Cardiac injury can occur early or later in the course of COVID-19 infection and recurrent episodes of cardiac injury may occur, with the latter events being associated with worse outcome (46). Importantly, more severe cardiac injury is associated with higher levels of TnI, greater need for ICU admission, and higher mortality (18).

The potential etiologies of COVID-related acute myocardial injury include i) ACS due to plaque rupture or thrombosis (Type I myocardial infarction), or supply-demand mismatch (Type II myocardial infarction), or ii) injury due to disseminated intravascular coagulation or non-ischemic injury, associated with myocarditis, stress-induced cardiomyopathy, cytokine release or acute pulmonary embolism (47). Cardiac injury is associated with more severe disease and a worse prognosis (43).

Interestingly, one hypothesis-generating study from the US, which mirrored similar studies from other countries, such as European countries, as well as Australia and New Zealand, indicated that most metropolitan cities noted a marked increase in out-of-hospital cardiac arrest, early in 2020, which generally paralleled the local prevalence of COVID-19 infections (48). It was suggested that this was likely due to the SARS-CoV-2 virus, but that these deaths could not be reported as being due to COVID-19 as testing was not done on most patients before they died. The study also did not attempt to address clinical details, such as data on cardiac events.

### Acute Myocardial Infarction/Acute Coronary Syndrome

The topic of COVID-19 and acute myocardial injury/ACS has been extensively reviewed (47). Among the common findings are firstly, that there is an increased risk of myocardial injury and infarction in patients with COVID-19 infection, especially in those patients with underlying cardiovascular comorbidities and/or risk factors. Secondly, there is a range of differential diagnoses with regard to the cause of myocardial injury, as noted above. Thirdly, lower rates of hospitalization have been noted worldwide for acute coronary syndrome during the COVID-19 pandemic, most likely because of patients’ hesitancy to present to hospital, as well as misdiagnosis. Lastly, lack of preparation and standardized protocols for early management of ACS, in the setting of protection of healthcare professionals and the environment, have potentially led to delayed treatment of ACS.

A recent study from Sweden, indicated that when day one of the infection was excluded, the odds ratio for an AMI in the two weeks following COVID-19 infection was 3.41 [95%CI 1.58-7.36] and when day 0 was included it was 6.61 [95% CI 3.56-12.20]. The authors suggested that AMI (and ischemic stroke) represented a part of the clinical picture of COVID-19 infection, highlighting the need for prevention by vaccination (49).

In one registry-based retrospective analysis of hospitalized patients with COVID-19 who suffered an AMI, the overall mortality was 22.8% (50). The deceased were older than survivors, and patients with hypertension, worsening renal function and higher cardiac troponin T and C-reactive protein levels were more likely to die. Importantly, one observational study documented that occult infections with SARS-CoV-2 (and influenza) occurred in patients presenting with AMI, but without COVID-19 symptoms (13% of cases) and while there was no difference in mortality in those with and without COVID-19 infection, the former group had a shorter time to a recurrent cardiovascular event (51).

Two studies investigated the clinical course and outcome of patients with AMI, with and without COVID-19 infection. The one study analyzed patients in a prospective COVID-ACS international registry, using a pre-COVID-19 cohort as the control group (52), while the other compared COVID-19-negative and -positive cases admitted to hospital with AMI early in the pandemic (53). The former study documented that patients with COVID-19 and ACS presented later and had an increased in-hospital mortality compared to the pre-COVID-19 cohort, and that there was a greater rate of cardiogenic shock, which was a major contributor to the poorer outcome. The latter study documented that patients with COVID-19 and AMI were older, had more comorbidities, and had a higher hospital mortality, compared to those without COVID-19.

Some authors have restricted their analyses to those cases with ST elevation myocardial infarction (STEMI) (54–56). The first study compared patients with confirmed COVID-19 and STEMI with an age- and sex-matched STEMI group prior to COVID-19 (54). Patients with COVID-19 infection were more likely to present with cardiogenic shock (18%), but less likely to have angiography (78%) compared to the control group. The primary outcome was a composite of in-hospital death, stroke, recurrent AMI and repeat unplanned revascularization, which occurred more commonly in the COVID-19-positive group compared to controls. The second study showed a significant increase in in-hospital mortality, stent thrombosis and cardiogenic shock after percutaneous revascularization in those patients with STEMI and COVID-19 infection compared with a contemporaneous group of non-COVID STEMI patients (55). The third study indicated that STEMI patients admitted during the first wave of COVID-19 infections in Israel, had a longer ischemic time, which translated into more severe disease on hospital admission and a higher in-hospital adverse event rate than observed in STEMI cases admitted in a corresponding period in 2018 (57).

Interestingly a literature review of COVID-19 patients who had STEMI, in the early part of the pandemic, documented that 17% of cases were due to non-obstructive coronary artery disease, which was associated with a mortality of 30% (similar to those with obstructive coronary artery disease); however, such data were based on case reports and case series only (57). The role of thrombosis, including extensive and multi-vessel thrombosis, irrespective of the presence or absence of atherosclerotic plaques has been described, and is reviewed elsewhere (58, 59).

Other studies have analyzed predominantly Type II myocardial infarction in patients with COVID-19 (60). These studies indicate that such cases may be caused by an imbalance between oxygen supply and demand, possibly due to hypoxia, increased heart rate, systemic inflammation and/or decompensated cardiac failure, and are associated with high in-hospital mortality rates.

Clearly, a number of studies, some described above, suggest that there has been a delay in health-seeking behavior, primarily due to fear of contracting SARS-CoV-2 infection. This may account for the apparent decrease in prevalence of AMI during COVID-19 and be associated with a delay in medical intervention, resulting in an alarming rate of rare complications from AMI (48, 52, 61).

## The Role of Platelets in Ischemic Cardiac Disease

Platelets play an important role in the development of thrombo-inflammatory conditions and complications and are thus recognized as critical participants in the development of AMI (62). Platelet activation is a complex and multistep process (which is beyond the scope of the current review), principally mediated by specific platelet receptors that control adhesion and activation (62). Notable amongst these are the platelet collagen receptor: Fc receptor: γ-chain (FcR γ) co-expressed with glycoprotein (GP) VI. Patients with ACS have been reported to express higher numbers of GPVI receptors (63), have altered GPVI signaling (64) and an increased aggregation response (64).

Various platelet surface activation markers, such as CD62P (P-selectin), CD40L, platelet factor 4 and GP IIb/IIIa, as well as biological markers, primarily platelet micro-particles, have been associated with inflammation, atherosclerosis, and thrombosis (65). Platelet-monocyte aggregates, which persist in peripheral blood for longer than the surface markers and are therefore viewed as more sensitive markers of *in vivo* platelet activation (65), are an early marker of acute MI and have also been linked to myocardial no-reflow in STEMI patients (66, 67). Indirect markers of platelet activation, such as increased mean platelet volume (68), as well as higher levels of von Willebrand factor (69), and serotonin (70), have also been observed during acute MI.

## Platelet Interactions With SARS-CoV-2

In addition to expression of the low-affinity receptor for the Fc fragment of immunoglobulin G, which may promote viral entry, platelets also express TLR7, as mentioned above, which enables these cells to interact with viral single-stranded RNA (71, 72). Both of these mechanisms, as well as others (73), may contribute to viral persistence and hyperreactivity of platelets. In the case of ACE2, the major SARS-Cov-2 cell entry receptor and its associated transmembrane serine protease 2 (TMPRSS2), expression of this viral receptor and enzyme by platelets has been reported by some (74), but challenged by others (75). Because a direct role, if any, for the spike (S) protein of SARS-Cov-2 in mediating platelet hyperreactivity and thrombocytopenia in severe disease remains to be established, this section of the review is largely focused on severe COVID-19 that is associated with endothelial infection, activation and dysfunction as significant contributors to platelet overdrive and development of vascular disease.

## Platelet Interaction With Inflamed/Damaged Endothelium

Although platelets do not normally interact with intact vascular endothelium, they nevertheless play a critical role in maintaining vascular homeostasis *via* continuous monitoring of endothelial integrity. This is accomplished by preventing and repairing vascular leaks such as those associated with leukocyte extravasation (76). However, in the setting of the excessive endothelial activation and dysfunction that occur during serious SARS-CoV-2 infection, platelets acquire a more aggressive phenotype. Notwithstanding platelet activation mediated by thrombin and collagen, as well as autocrine activation by adenosine 5’-monophosphate and thromboxane A_2,_ this is augmented by interaction of platelet Toll-like receptor 7 (TLR7) with viral single stranded RNA. Platelet activation is characterized by increased expression of CD62P and tissue factor, as well as platelet/leukocyte aggregate formation, which contribute significantly to the pathogenesis of thrombotic disease (77–79).

In this setting, the interaction of platelets with stressed vascular endothelium involves most of the adhesive mechanisms that also maintain vascular homeostasis. However, the transition from homeostatic regulation to vascular disease is determined by the extent of endothelial dysfunction and the associated increase in the intensity and duration of platelet adhesion and activation that drives the recruitment of highly reactive neutrophils and monocytes.

Mechanisms that promote the adhesion of platelets to inflamed vascular endothelium are numerous and these have been extensively reviewed elsewhere (80, 81). Prominent among these are initial adhesive events that promote “rolling” of platelets along endothelium, particularly the interaction of the major platelet membrane receptor, GP1b (CD42)-1X-V with von Willebrand factor released from endothelial cells (ECs). This type of rolling action is reinforced by interactions that involve upregulated expression of CD62P, present on both cell types, with its counter receptor, P-selectin glycoprotein ligand-1 (PSGL-1), also expressed on both cell types, albeit weakly in the case of platelets (81).

Stabilization of platelet/EC interactions requires the cooperation of two key integrins and three endothelial-associated, pro-adhesive molecules. These are the integrins, α_IIb_β_3_ and α_v_β_3,_ expressed on platelets and ECs, respectively, and the proteins, fibrinogen and fibronectin, as well as the glycoprotein, von Willebrand factor, which act as bridges linking the platelet and EC integrins to achieve firm binding. This is intensified *via* the fibrinogen-dependent interactions of α_IIb_β_3_ with intercellular adhesion molecule-1 (ICAM-1; CD54) expressed on ECs, as well as by the association of EC-bound GP1b-IX-V/von Willebrand factor with platelet α_IIb_β_3_ (81).

In addition to these platelet-endothelium interactions, other prominent mechanisms by which prolonged platelet activation poses risks to cardiovascular health include the following:

• microvascular occlusion due to formation of large intravascular homotypic aggregates of platelets, as well as heterotypic aggregates of platelets with neutrophils and monocytes (78, 82);
• platelet-driven systemic inflammatory responses associated with activation of neutrophils and monocytes/macrophages, resulting in excessive production of proinflammatory cytokines, especially interleukin (IL)-6, as well as induction of intense intracellular oxidative stress (83, 84);
• excessive platelet-driven intravascular formation of neutrophil extracellular traps (NETs) that contribute to organ damage and dysfunction (85, 86);
• release of prothrombotic/procoagulant factors (factor V, prothrombin, fibrinogen, von Willebrand factor) from platelet α-granules (87);
• platelet-driven active and passive expression of tissue factor by monocytes and neutrophils, respectively (78, 88).

These platelet-driven inflammatory mechanisms are summarized in **Table 3**.

**Table 3:** Mechanisms by which activated platelets contribute to vascular and organ damage during severe COVID-19 infection.

Mechanism	Refs
• Formation of large intravascular homotypic (platelets) and heterotypic (platelets/neutrophils/monocytes) aggregates that result in microvascular occlusion	(78, 82)
• Initiation and perpetuation of systemic inflammatory responses that drive excessive production of proinflammatory cytokines and severe oxidative stress	(83, 84)
• Drive the intravascular formation of obstructive neutrophil extracellular traps	(85, 86)
• Release prothrombotic/procoagulant factors contained in α-granules	(87)
• Initiate synthesis and release of tissue factor by platelet-activated monocytes	(78, 88)

## Platelet-Mediated Endothelial Damage and Dysfunction

In addition to the direct proinflammatory/prothrombotic and cytotoxic effects of the SARS-Cov-2 S protein on vascular endothelium, as well as impairment of the renin-angiotensin-aldosterone system (83), prolonged platelet activation in severe COVID-19 infection also amplifies endotheliopathy (89) In this context, a very recent study by Barrett et al. described potential mechanisms by which activated platelets exacerbate endothelial activation and damage (90). Using a strategy based on integration of endothelial cells and platelets isolated from patients hospitalized with COVID-19, transcriptomic analysis of mRNA expression extracted from endothelial cells exposed to platelet releasate revealed alterations in processes involved in maintenance of tight junction barrier integrity, coagulation and inflammation, characteristic of an “inflammatory, hypercoaguable phenotype” (90).

Systematic genetic analysis enabled the authors to identify the genes encoding the calcium-binding proteins, SA100A8 and SA100A9, also known as myeloid-related proteins (MRP) 8 and 14, respectively, as being the most prominent platelet-derived mediators of endothelial activation. These proteins are stored in platelet granules and dimerize to form the potent, proinflammatory protein, calprotectin (MRP8/14). Interacting with CD36 on vascular endothelium, calprotectin weakens cell-cell interactions and initiates release of the proinflammatory cytokines, IL-6, and IL-8 (90–92).

Importantly, the systemic levels of MRP8/14 were found to be significantly higher in the cohort of patients hospitalized with COVID-19 relative to those of control subjects and were associated with development of thrombosis and other adverse events (90).

Very recently, Bye et al. described another mechanism operative in patients with severe COVID-19 by which platelets may augment endothelial activation and dysfunction (93). In this setting, platelets that have adhered to SARS-CoV-2-infected endothelial cells *via* von Willebrand factor make contact with surface-bound immune complexes formed between the S protein and heavily Fc region-galactosylated immunoglobulin G antibodies, which effectively bind to and activate the platelet FcγRIIA receptor (93).

## Endotheliitis due to Serious SARS-CoV-2 Infection

Notwithstanding the involvement of cells of the innate and adaptive immune systems, there is increasing recognition of the key role played by the vascular endothelium in the development of the prothrombotic state and hypercoagulability that precede the occurrence of arterial and venous complications in patients with severe SARS-CoV-2 infection, particularly those with preexisting, procoagulant tendencies (94–97). Of the 29 SARS-CoV-2 proteins, it is the S protein in particular and, to a lesser extent, the nucleocapsid protein (NP) that drive virus-mediated endothelial damage. Of these two viral proteins, the former is cytotoxic, causing loss of endothelial structural integrity, while the latter induces a proinflammatory endothelial phenotype. Importantly, ECs do not express TLR7, excluding putative proinflammatory interactions with viral single-stranded RNA (98).

### Spike Protein-Mediated Endothelial Dysfunction

This viral protein consists of two subunits, S1 and S2. These spike proteins bind initially to endothelial surface glycosaminoglycans, enabling recognition of, and interaction with, their natural cellular counter receptor, ACE2, which is expressed on several types of host cells, including vascular endothelium (99). Physical binding of ACE2 to the S protein necessitates proteolytic cleavage at the S1/S2 sites by the cellular enzyme, TMPRSS2. These events precede, and are a prerequisite, for membrane fusion and viral entry. Thereafter S protein-mediated endothelial dysfunction results from at least two distinct mechanisms, both of which are dependent on viral infection of ECs, which results from binding of the S1 region to ACE2.

Firstly, and very recently, Lei et al. engineered an RNA-free pseudovirus variant of SARS-CoV-2 that was comprised of an inert outer shell impregnated with the S protein that enabled efficient attachment to ACE2 (99). Intratracheal administration of this variant of SARS-CoV-2 to Syrian hamsters mimicked many of the features of the pulmonary damage associated with natural SARS-CoV-2 infection. These changes included downregulation of endothelial expression of ACE2 that was due to decreased stability of the virus receptor that resulted from impaired phosphorylation of the ACE2 Ser-680 amino acid residue by the endothelial enzyme, AMP-activated protein kinase (AMPK) (99). This event, in turn, targeted the dephosphorylated variant of ACE2 for proteolytic degradation by a pathway driven by the oncoprotein, MDM2 (murine double minute 2) that mediates ubiquitination of unstable ACE2 (99, 100). In addition, dysregulation of endothelial protein phosphorylative activity also resulted in interference with the activity of endothelial nitric oxide synthase (eNOS) (99).

The authors replicated these findings in a series of *in vitro* experiments in which isolated pulmonary arterial ECs were infected with the SARS-CoV-2 pseudovirus (99). In addition, they also observed that exposure of the ECs to recombinant S1 protein caused mitochondrial dysfunction and EC fragmentation, seemingly because of intense intracellular oxidative stress associated with loss of ACE2. Based on their findings, the authors concluded that SARS-CoV-2 S1 protein-mediated loss of endothelial ACE2 “may exacerbate endothelial dysfunction, leading to endotheliitis” (99).

The aforementioned findings are largely in keeping with those of another very recent study reported by Raghavan et al. (101). Using an *in vitro* cell culture-based approach, these authors investigated the effects of exposure to recombinant S1 protein on the structural integrity of primary cultures of mouse brain ECs isolated from non-diabetic and diabetic mice (101). The authors measured the pro-adhesive activities of several types of EC junctional adhesion molecules [vascular endothelial (VE) cadherin; junctional adhesion molecule-A (JAM-A) that regulates tight junctions; the gap junction protein, connexin-43; PECAM-1 (CD31) also highly expressed at EC intercellular junctions]. Following exposure to the S1 protein, the cellular levels of all four junctional adhesion molecules decreased significantly, resulting in disruption of the endothelial barrier, an effect that was most evident with ECs from diabetic mice (101). Mechanistically, S1 protein-mediated endothelial disruption appeared to result from an increased association of the junctional adhesion molecules with the membrane-associated protein, Rab5a, which is a GTPase regulator of intracellular vesicular transport that regulates the movement of membrane proteins between the plasma membrane and early endosomes (101, 102). In the clinical setting of COVID-19, loss of barrier function results not only in detachment of ECs that are detected as circulating cells floating in blood (103), but also exposure of the arterial sub-endothelium with the attendant risk of myocardial infarction and stroke.

Notwithstanding involvement in infection and death of ECs by SARS-CoV-2, the role of ACE2, if any, in S1 protein-mediated junctional adhesion molecule dysfunction does, however, remain to be established.

### Spike Protein and Complement-Mediated Dysfunction of Endothelial Cells

Recently, Yu et al., albeit also using an *in vitro*-based approach, demonstrated that treatment of eukaryotic cells with either the S1 or S2 recombinant proteins, but not NP, initiated endothelial cytotoxicity *via* activation of the alternative complement pathway (104). The authors used an engineered *PIGA*null TF1 cell line that does not express ACE2 for their studies. This is an erythroleukemic cell line genetically depleted of phosphatidylinositol glycan class A (*PIGA*), which is the precursor of glycosylphosphatidylinositol (GPI). This glycophospholipid anchors the complement regulatory proteins, CD55 (inactivates the C3 convertases of the alternative and classical pathways) and CD59 (attenuates the lytic activity of the membrane attack complex). Cytotoxicity of both the S1 and S2 proteins occurred in the presence of normal human serum measured using a colorimetric assay based on cellular metabolic activity (104). The involvement of the alternative, as opposed to the classical pathway of complement activation, was demonstrated according to the presence of increased levels of fragment Bb (derived from activation of Factor B), as well as by the protective effects of inhibition of Factor D and by the addition of Factor H (104). Although remaining to be conclusively established, the authors speculate that the S1 and S2 proteins initiate activation of the alternative complement pathway by interfering with the activity of regulatory Factor H (104).

Although of potential pathophysiological significance, studies of this type need to be repeated using endothelial cell lines with intact complement regulatory surface proteins, as well as expression of ACE2.

### Activation of the NLRP3 Inflammasome by the S and Viroporin SARS-CoV-2 Proteins

The S protein of SARS-CoV-2 has also been shown to activate the proinflammatory/pro-pyroptosis NLR family pyrin domain-containing protein 3 (NLRP3) inflammasome, seemingly by a pro-oxidative mechanism in various types of eukaryotic cells, including small human hematopoietic and EC precursor cells, as well as macrophages (105, 106). In addition, the SARS-CoV-2 small membrane permeability-inducing protein, viroporin, may also potentiate activation of the NLRP3 inflammasome *via* a mechanism involving efflux of K^+^ (107). Importantly, the NLRP3 inflammasome is present in vascular endothelium, activation of which triggers endothelial dysfunction and death (108). Although seemingly unexplored, putative activation of the EC NLRP3 inflammasome may also contribute to the development of SARS-CoV-2-mediated endotheliitis.

### Nucleocapsid Protein

The NP of SARS-CoV-2 is a 45.6 kDa (theoretical) multivalent RNA-binding protein that initiates activation of ECs by mechanisms that are non-cytotoxic and do not involve ACE2 (109). In this context, Qian et al. recently investigated the effects of exposure of five different types of human primary EC cultures (pulmonary vascular, umbilical vein, aortic, coronary artery and dermal microvascular) to recombinant NP (0.05–1 micrograms/milliliter) for 4–24 hours *in vitro* (109). This resulted in dose- and time-related increased expression of the endothelial adhesion molecules, intercellular adhesion molecule-1 (ICAM-1) and vascular adhesion molecule-1 (VCAM-1), that promoted adherence of human primary monocytes (109). Mechanistically, NP-mediated endothelial activation involved interaction of the viral protein with TLR2 expressed on ECs. This event, in turn, triggered activation of intracellular signaling mechanisms driven by nuclear factor kappa B (NF-κB) and mitogen-activated protein kinase (MAPK), resulting in transcription of the genes encoding ICAM-1 and VCAM-1 (109).

Although indicative of a proinflammatory activity of the SARS-CoV-2 NP that may contribute to the endotheliitis of advanced COVID-19 infection, independent confirmation of these findings, as well as extrapolation to the pathogenesis of thrombosis in small vessels, is necessary.

These various mechanisms by which the SARS-Cov-2 spike protein, viroporin and NP promote endothelial damage and dysfunction are summarized in **Table 4**.

**Table 4:** SARS-Cov-2 proteins that mediate endothelial cell dysfunction.

Protein	Mechanisms	Refs
Spike protein	• Degradation of ACE2 • Decreased activity of eNOS • Mitochondrial dysfunction and cellular oxidative stress	(99)
	• Displacement of endothelial junctional adhesion molecules	(101)
	• Cytotoxicity due to activation of the alternative complement pathway	(104)
	• Possible activation of the endothelial NLRP3 inflammasome potentiated by viroporin	(105–108)
Nucleocapsid protein	• Upregulation of expression of the endothelial adhesion molecules, ICAM-1 and VCAM-1, resulting in recruitment of inflammatory cells	(109)

ACE, acetylcholine esterase; eNOS, endothelial nitric oxide synthase; NLRP3, NLR family pyrin domain-containing protein 3; ICAM-1, intercellular adhesion molecule-1; VCAM-1, vascular cell adhesion molecule-1.

Given the very recent interest in the involvement of the vascular endothelium in the pathogenesis of COVID-19, only a few reports have described relationships between disease severity and outcome with elevations in systemic biomarkers of endothelial activation and dysfunction such as von Willebrand factor and thrombomodulin (110–112). The paucity of reports focused on this important topic underscores the necessity for more intensive investigation. Novel platelet-directed therapies currently under investigation include the P-selectin-targeted monoclonal antibody, crizanlizumab (113).

## Adverse Consequences of a Damaged Endothelium

**Figure 1:**
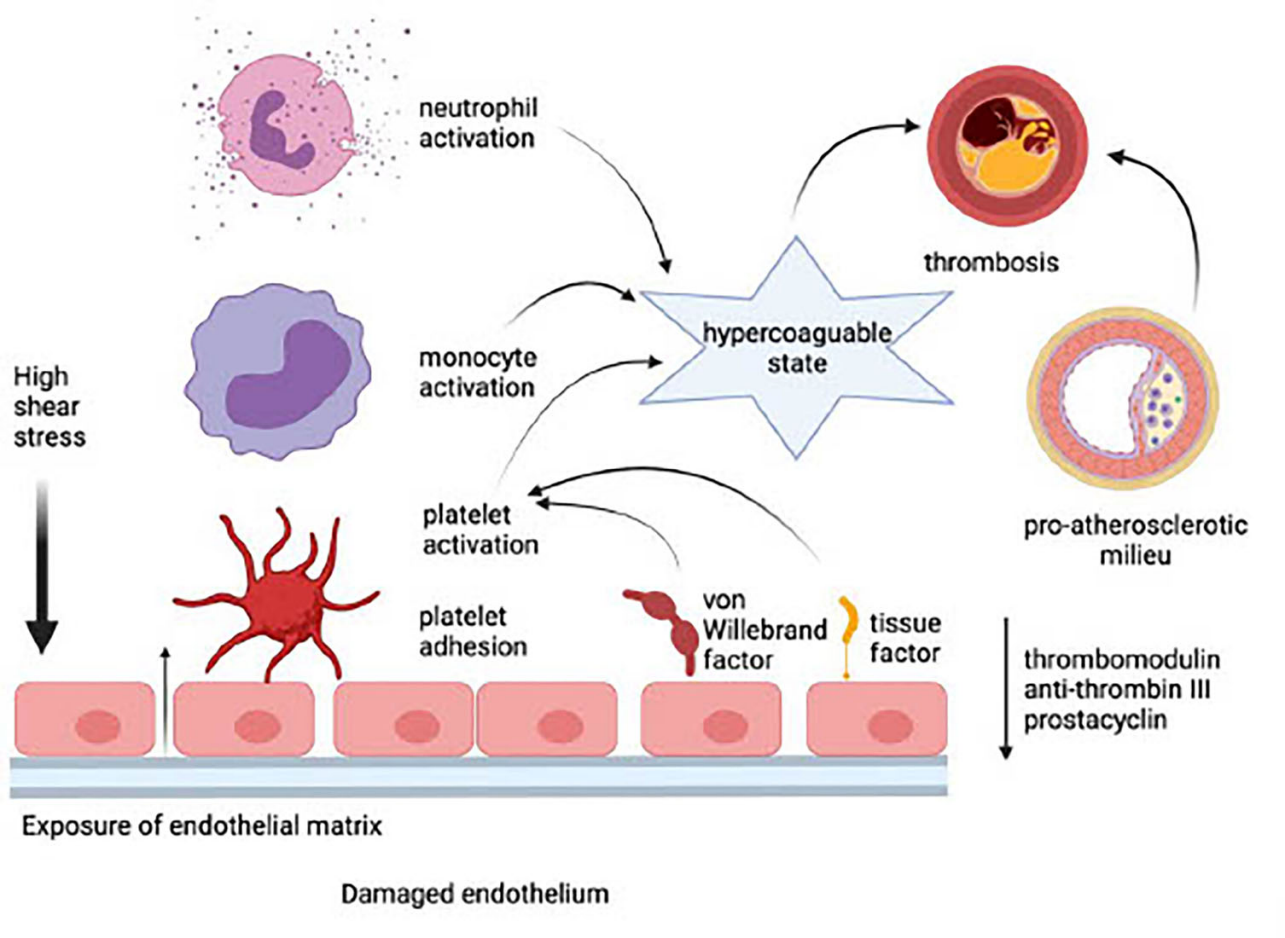
Adverse consequences of a damaged endothelium (created in Biorender). In the setting of high shear stress, exposure of the sub-endothelial extracellular matrix leads to the loss of endothelial cell-derived anti-coagulant proteins such as thrombomodulin and anti-thrombin III, as well as the prostanoid, prostacyclin. Platelets are activated via endothelial leakage of von Willebrand factor and by increased synthesis and expression of tissue factor by endothelial cells. Platelet accumulation and interaction with monocytes and neutrophils potentiate a hypercoagulable state, which, in the presence of a pre-atherosclerotic milieu, causes thrombosis and predisposes for development of myocardial infarction and stroke.

Endothelial damage precedes the adhesion and activation of platelets, neutrophils, and monocytes. This event, in turn, results in the development of a procoagulant state due to the loss of EC-derived anti-coagulant proteins such as thrombomodulin and anti-thrombin III, as well as the prostanoid, prostacyclin. These procoagulant mechanisms are intensified, in turn, by potentiation of platelet activation *via* endothelial leakage of von Willebrand factor and by increased synthesis and expression of tissue factor by ECs. Inflammatory events that occur on an essentially intact endothelium in the setting of a low shear stress predispose for the development of venous thrombosis (114). In the case of arterial thrombosis, disruption of the endothelial barrier in the setting of high shear stress, exposure of the sub-endothelial extracellular matrix, platelet accumulation, and, in particular, the existence of a pre-atherosclerotic milieu, are likely to predispose for development of MI and stroke. These processes are depicted in **Figure 1**.

## Conclusion

COVID-19 is a systemic disease affecting various systems, including the cardiovascular system, where it leads to significant mortality. While our understanding of the pathogenesis of COVID-19 and associated complications is evolving, evidence is emerging about the very important role of platelets *per se* together with endotheliitis and its attendant role in platelet recruitment and activation. These events create a SARS-CoV-2-driven cycle of intravascular inflammation and coagulation, which contributes significantly to a poor clinical outcome in patients with severe disease. Clearly, the role of platelet-endothelium interactions in the pathogenesis of COVID-19 needs further intense exploration to enable optimization of the treatment of the infection and its complications with the ultimate goal of improving outcomes.

## Author Contributions

TR, RA, and CF contributed equally to the conceptualization and compilation of the manuscript, while all four authors (TR, RA, PM, and CF) were involved in reviewing and finalizing the manuscript and approved its submission. All authors contributed to the article and approved the submitted version.

## Conflict of Interest

The authors declare that the research was conducted in the absence of any commercial or financial relationships that could be construed as a potential conflict of interest.

## Publisher’s Note

All claims expressed in this article are solely those of the authors and do not necessarily represent those of their affiliated organizations, or those of the publisher, the editors and the reviewers. Any product that may be evaluated in this article, or claim that may be made by its manufacturer, is not guaranteed or endorsed by the publisher.
